# Evaluation of Canonical siRNA and Dicer Substrate RNA for Inhibition of Hepatitis C Virus Genome Replication – A Comparative Study

**DOI:** 10.1371/journal.pone.0117742

**Published:** 2015-02-23

**Authors:** Bruno Carneiro, Ana Cláudia Silva Braga, Mariana Nogueira Batista, Mark Harris, Paula Rahal

**Affiliations:** 1 Genomics Study Laboratory, Sao Paulo State University, IBILCE, São José do Rio Preto, SP, Brazil; 2 School of Molecular and Cellular Biology, Faculty of Biological Sciences, University of Leeds, Leeds, LS2 9JT, United Kingdom; University of Montreal Hospital Research Center (CRCHUM), CANADA

## Abstract

Hepatitis C virus (HCV) frequently establishes persistent infections in the liver, leading to the development of chronic hepatitis and, potentially, to liver cirrhosis and hepatocellular carcinoma at later stages. The objective of this study was to test the ability of five Dicer substrate siRNAs (DsiRNA) to inhibit HCV replication and to compare these molecules to canonical 21 nt siRNA. DsiRNA molecules were designed to target five distinct regions of the HCV genome – the 5’ UTR and the coding regions for NS3, NS4B, NS5A or NS5B. These molecules were transfected into Huh7.5 cells that stably harboured an HCV subgenomic replicon expressing a firefly luciferase/neoR reporter (SGR-Feo-JFH-1) and were also tested on HCVcc-infected cells. All of the DsiRNAs inhibited HCV replication in both the subgenomic system and HCVcc-infected cells. When DsiRNAs were transfected prior to infection with HCVcc, the inhibition levels reached 99.5%. When directly compared, canonical siRNA and DsiRNA exhibited similar potency of virus inhibition. Furthermore, both types of molecules exhibited similar dynamics of inhibition and frequencies of resistant mutants after 21 days of treatment. Thus, DsiRNA molecules are as potent as 21 nt siRNAs for the inhibition of HCV replication and may provide future approaches for HCV therapy if the emergence of resistant mutants can be addressed.

## Introduction

Infection with hepatitis C virus (HCV) is a worldwide public health problem, a major cause of liver cirrhosis and hepatocellular carcinoma, and has been considered the leading indication for liver transplantation [1]. Approximately 170 million people are chronically infected with HCV worldwide, and studies in the USA found that deaths caused by HCV infection exceeded those resulting from HIV infection [2]

HCV is an RNA virus and member of the Flaviviridae family and the Hepacivirus genus. The virus has a 9.6 kb single-stranded positive-sense genome that encodes a single polyprotein comprising approximately 3000 amino acids flanked by 5’ and 3’ untranslated regions (UTRs). Translation is driven in a cap-independent fashion by an internal ribosome entry site (IRES), and both viral and host proteases cleave the polyprotein to yield 10 structural (Core, E1 and E2) and non-structural (p7, NS2, NS3, NS4A, NS4B, NS5A and NS5B) proteins.

The infection initially causes acute hepatitis, which is often subclinical and may develop into a chronic condition. The evolution to chronicity occurs in approximately 85% of cases [3], and among these chronic patients, 70% develop liver pathology. Five to twenty per cent of these pathologies are liver cirrhosis [4], and 1 to 5% of patients die from cirrhosis or hepatocellular carcinoma. Approximately 30–50% of patients develop hepatocellular carcinoma after approximately 10 years of infection [5].

Until recently the standard of care for chronic HCV infection was pegylated interferon (peg-IFN) and ribavirin (RBV) [6]. The development of novel direct acting antivirals (DAA) targeting the NS3 protease (e.g. boceprevir, telaprevir or simeprevir), NS5A phosphoprotein (e.g. daclatasvir) and NS5B polymerase (e.g. sofusbuvir) has revolutionised treatment [6]. The new treatment regimens have dramatically improved the sustained virologic response (> 80%) but, severe side-effects and the high-cost are still a problem on HCV therapy [7]. Because of that, several new drugs are being tested at the time. However, because HCV is the most variable virus known to man, the application of selective pressure via drug treatment will undoubtedly lead to resistance [8,9]. Therefore, continuing to identify new drugs and more effective treatments is important. In this regard, RNA interference (RNAi) has been demonstrated both *in vitro* and *in vivo* to have the potential to treat various viral infections.

RNA interference is a process of post-transcriptional gene silencing that has been identified in all eukaryotes [10,11]. Since its first report, this mechanism has been found to be useful both as a molecular biology tool for the study of gene function and as a therapeutic agent [12,13]. A key component of the RNAi pathway is the RNA-induced silencing complex (RISC), which is responsible for the cleavage of mRNA in a sequence-specific fashion. The specificity of this reaction is provided by a 21 nt antisense strand incorporated into the RISC complex. This antisense strand originates from the digestion of double stranded RNA (dsRNA) by DICER endonuclease [14]. Synthetic 21 nt siRNAs can be introduced into cells and can selectively suppress a specific gene of interest. A number of reports have shown that RNA interference can efficiently inhibit HCV replication via different methodologies [15–17].

DICER substrate siRNAs (DsiRNAs) are 25/27-nt-long asymmetrical double stranded RNAs that can inhibit a specific mRNA sequence without activating the IFN pathway [18]. These DsiRNA are recognised by DICER enzymes and digested into smaller 21 nt dsRNA fragments, which are then recognised and incorporated by the RISC complex to finally degrade the targeted mRNA. Recent studies have shown that DsiRNAs are more potent than conventional synthetic siRNAs [19,20].

A major drawback for therapy with RNAi for an RNA virus is the RNA-dependent RNA polymerase (RdRp) responsible for viral genome replication. RdRp has a high error rate due to a lack of proofreading activity; therefore, the mutation rates for these viruses are relatively high [21]. The long-term treatment of cells infected with HCV or of those harbouring an SGR with RNAi molecules induced the selection of resistant viral quasi-species, which reduced the treatment efficiency [22].

In this present study, we developed five DsiRNA molecules directed to the HCV genome and showed that these molecules could efficiently inhibit virus replication. Contrary to previous studies, we also demonstrated that DsiRNA and siRNA have the same potency for HCV inhibition. Moreover, we did not observe any difference in the frequency of resistant mutants that were selected after the treatment of subgenomic replicon (SGR)-harbouring cells with these RNAs after 21 days.

## Materials and Methods

### HCV replicon constructs

SGR-Feo-JFH-1 is a bicistronic subgenomic replicon [23] based on the genotype 2a JFH-1 virus isolate that contains a firefly luciferase gene fused to a neomycin resistance gene (Feo). FL-J6/JFH-5′C19Rluc2AUbi is a monocistronic full-length virus construct [24] based on the sequence of J6 (genotype 2a) and JFH-1 (genotype 2a), which expresses the *Renilla* luciferase gene. SGR-Feo-JFH-1 was used to produce a stable Huh7.5 cell line; FL-J6/JFH-5′C19Rluc2AUbi was used to produce HCVcc particles.

### Mammalian cell culture

Huh7.5 cells [25] were cultured in DMEM (Sigma Aldrich, St. Louis, MO, USA) supplemented with 10% (v/v) heat-inactivated foetal bovine serum (Cultilab, Campinas, SP, Brazil), 1x nonessential amino acids, 100 U/ml penicillin and 100 μg/ml streptomycin (Life Technologies, Carlsbad, CA, USA) at 37°C in a 5% CO_2_ humidified incubator. Stable cell lines were maintained with 800 μg/ml G418 (Sigma Aldrich).

### RNA transfection of Huh7.5 cells

RNA was transcribed from plasmid templates *in vitro* as previously described [26]. Huh7.5 cells were electroporated following the protocol described by Amako et al. [27]. To generate stable cell lines, 1 μg of transcribed RNA was transfected with a DMRIE-C reagent (Life Technologies) following the manufacturer’s instructions. Forty-eight hours after transfection, 800 μg/mL G418 (Sigma Aldrich) was added to the culture medium. Colony formation was observed after four weeks.

### DsiRNA and siRNA targeted to the HCV genome

Five Dicer substrate siRNAs (DsiRNAs) that were 25/27 nt in length were designed to target five distinct regions of HCV mRNA: 5’UTR, NS3, NS4B, NS5A and NS5B. The DsiRNA molecules (IDT) were designed according to the algorithm described by Kim et al. [18]. DsiRNA molecules are asymmetrical, the sense strand is 25 nt long with the last two bases being DNA and the antisense strands are composed of 27 RNA bases. Those modifications are necessary to help Dicer processing and incorporation to the correct strand to RISC complex [28]. Two 21 nt siRNA molecules were also designed to target the same sequence in the NS5B or NS4B coding region as the corresponding DsiRNA. A negative control DsiRNA with no homology to any human gene or to the HCV genome was also utilised (NC1—IDT). For canonical siRNA analyses, we utilised a negative siRNA control that also lacked homology to HCV or any human gene (Negative control 1 siRNA—Life Technologies).

### DsiRNA Transfection method

SGR-Feo-JFH-1-harbouring Huh7.5 cells were trypsinised, and 3 x 10^4^ cells/well seeded into a 24-well plate and incubated for 24 h prior to transfection. All transfections were carried out using the RNAi-specific cationic lipid formulation Lipofectamine RNAiMAX (Life Technologies) following the manufacturer’s instructions. The transfection efficiency was monitored via transfection with TYE 563 DS Transfection Control (IDT), a DsiRNA duplex labelled with a fluorescent dye.

### DsiRNA transfection in HCVcc-infected Huh7.5 cells

HCVcc containing supernatant was titrated using a focus-forming assay, and Huh7.5 cells were infected at a multiplicity of infection of 0.1. Twenty-four hours after infection, the cells were washed twice with PBS, fresh complete medium was added to the wells, and the cells were transfected with DsiRNA as described above. Forty-eight hours after infection, the cells were lysed and subjected to a luciferase assay. Alternatively, the cells were first transfected with DsiRNAs 24 h prior to infection with the infectious supernatant using the same multiplicity of infection.

### Selection of resistant mutations

To evaluate the ability of either siRNA or DsiRNA to induce the formation of resistant clones, the cells were transfected three times (5 days apart) with siRNA, DsiRNA or the negative control molecule (NC1). Cells were initially seeded on a 24 well plate and whenever the cells approached confluence, they were trypsinised and transferred onto a larger plate. The selection medium containing 1 mg/ml G418 (Sigma Aldrich) was changed every two days. After 21 days of treatment, the selection medium was removed and the colonies were harvested by adding Trizol reagent (Life Technologies).

### Sequencing of resistant colonies

The total RNA was extracted from surviving colonies using Trizol (Life technologies) according to the manufacturer's instructions. Five hundred nanograms of RNA was reverse-transcribed using the Maxima First Strand cDNA Synthesis Kit for RT-qPCR (Thermo Scientific, Waltham, MA, USA). Complementary DNA was amplified using primers flanking the target region of siRNA/DsiRNA and Phusion Hot Start II High-Fidelity DNA Polymerase (Thermo Scientific). The PCR product was subsequently cloned into a pBlunt vector (Thermo Scientific) and transformed into Z-competent *E. Coli* (Zymo Research). Nineteen clones were selected and submitted for sequencing using the BigDye Terminator (Life technologies), and the products were sequenced in an ABI 3130XL Sequencer (Life technologies).

### Luciferase assay

The cells were harvested by adding 100 μL of Passive Lysis Buffer (Promega). Twenty-five microlitres of cell lysate were transferred to a white 96-well plate. Next, 50 μL of Luciferase substrate (Luciferase Assay System, Promega or Renilla Luciferase Assay system) was automatically added, and the luminescence was read with a BMG plate reader (BMG Labtech GmbH, Ortenberg, Germany).

### Antibodies

The polyclonal sheep antiserum against HCV NS5A was described previously [29]. Monoclonal antibodies to glyceraldehyde-3 phosphate dehydrogenase (GAPDH; Abcam, Cambridge, MA, USA) were obtained commercially and used at concentrations recommended by the manufacturer; HRP- (Life Technologies) secondary antibodies were used for western blotting.

### Immunoblotting

The cells were lysed in Glasgow lysis buffer (GLB; 10 mM Pipes-KOH pH7.2, 120 mM KCl, 30 mM NaCl, 5 mM MgCl_2_, 1% Triton X-100 (Sigma), 10% glycerol) [29] supplemented with protease and phosphatase inhibitors (2 mM Na_3_VO_4_, 5 mM NaF, 5 mM Na_4_P_2_O_7_). The proteins were resolved by SDS/PAGE and transferred to a PVDF membrane (Millipore, Bedford, MA, USA).

### Northern blotting

Huh7.5 cells were transfected with different concentrations of a DsiRNA molecule and 16 hours after transfection cells were harvested and RNA extracted using Trizol. Approximately 30 ug of RNA was separated by 15% denaturing Urea Polyacrylamide gel electrophoresis and transferred to positively charged nylon membrane (Hybond N+, Amersham) using a semi-dry blotting device. RNA was fixed to membrane by UV crosslinking and pre-hybridized for 3 h with pre-hybridization buffer (7% SDS, 0.5% BSA, 200 mM Na_2_HPO_4_ (ph 7.0), 50 pmol/ml denaturated salmon sperm DNA). Hybridization was carried out at 39°C for 16 h with hybridization buffer (pre-hybridization buffer containing biotinylated probe at 50 pmol/ml). Hybridized membrane was washed twice with medium stringency washing buffer (1x SSC and 0.1% SDS), once with high stringency washing buffer (0.1x SSC and 0.1% SDS) and then incubated with blocking buffer (10% BSA, 0.1 M Tris/HCl pH 7.5, 0.1 M NaCl, 2 mM MgCl_2_, 0.05% Triton X-100) for 1 hour. Blocked membrane was then incubated with streptavidin-HRP conjugate (GE Healthcare, 1:3000) for one hour, washed three times with washing buffer A (0.1 M Tris/HCl pH 7.5, 0.1 M NaCl, 2 mM MgCl_2_, 0.05% Triton X-100) and one time with Buffer B (0.1 M Tris/HCl pH 9.5, 0.1 M NaCl,50 mM MgCl2). ECL HRP substrate [30] was added to the membrane and exposed for the appropriated time lengths on a chemiluminescent detection module (ChemiDoc XRS+).

### Data analysis

All experiments were performed using three technical replicates and at least two independent events. For the inhibition assays, the data were normalised to the values of the appropriate negative control. For cytotoxicity and off-target assays, the values were normalised to mock controls (cells treated with transfection reagent only). The data were analysed using Prism Graphpad v.5.0. The results were analysed using a one-way ANOVA at a significance level of p ≤ 0.05.

## Results

To develop molecules that could efficiently inhibit HCV replication, the sequence of the HCV genotype 2a isolate JFH-1 was screened using the bioinformatics tool RNAi Design from IDT SciTools (IDT). Based on the algorithm developed by Kim et al. [18], the program suggested twenty different targets in the HCV genome, which could theoretically effectively inhibit the replication of the virus. Five sequences were selected from these options to be synthesised and tested *in vitro*. The inclusion criteria for the study were the scores given by the software and the spatial separation of the sequences across the virus genome. The exact position and sequence of the DsiRNA molecules are summarised in Table 1.

**Table 1:** Information of DsiRNA and siRNA molecules.

Name	Target	Position ^a^	Sequence ^b^
DsiRNA 1	NS5B	8914–8940	S: 5’ CAACCAUAUGGGUUCGCAUGGUC**CT** 3’
			AS: 3’ AGGUUGGUAUACCCAAGCGUACCAGGA 5’
DsiRNA 3	NS5A	6991–7017	S: 5’ CCUGCACCACCCACAGCAACACC**TA** 3’
			AS: 3’ GUGGACGUGGUGGGUGUCGUUGUGGAU 5’
DsiRNA 5	NS4B	5758–5784	S: 5’ CCAGUACCACCAUCCUUCUCAAC**AT** 3’
			AS: 3’ CUGGUCAUGGUGGUAGGAAGAGUUGUA 5’
DsiRNA 7	NS3	4038–4064	S: 5’ GCUCCAACUGGCAGUGGAAAGAG**CA** 3’
			AS: 3’ UACGAGGUUGACCGUCACCUUUCUCGU 5’
DsiRNA 19	5’ UTR	42–68	S: 5’ CUGUGAGGAACUACUGUCUUCAC**GC** 3’
			AS: 3’ GGGACACUCCUUGAUGACAGAAGUGCG 5’
siD1	NS5B	8914–8934	S: 5’ CAACCAUAUGGGUUCGCAUGG 3’
			AS: 3’ AGGUUGGUAUACCCAAGCGUA 5’
siD5	NS4B	5758–5778	S: 5’ CCAGUACCACCAUCCUUCUCA 3’
			AS: 3’ CUGGUCAUGGUGGUAGGAAGA 5’
Probe	DsiRNA 1	-	5’ BTN—**ACTCCAACCATATGGGTTCGCAT** 3’

^a^ Position relative to HCV genotype 2a sequence, JFH-1 strain (AB047639);

^b^ S: sense strand, AS: antisense strand, bolded letters represents DNA bases, BTN—biotin.

**Fig 1:**
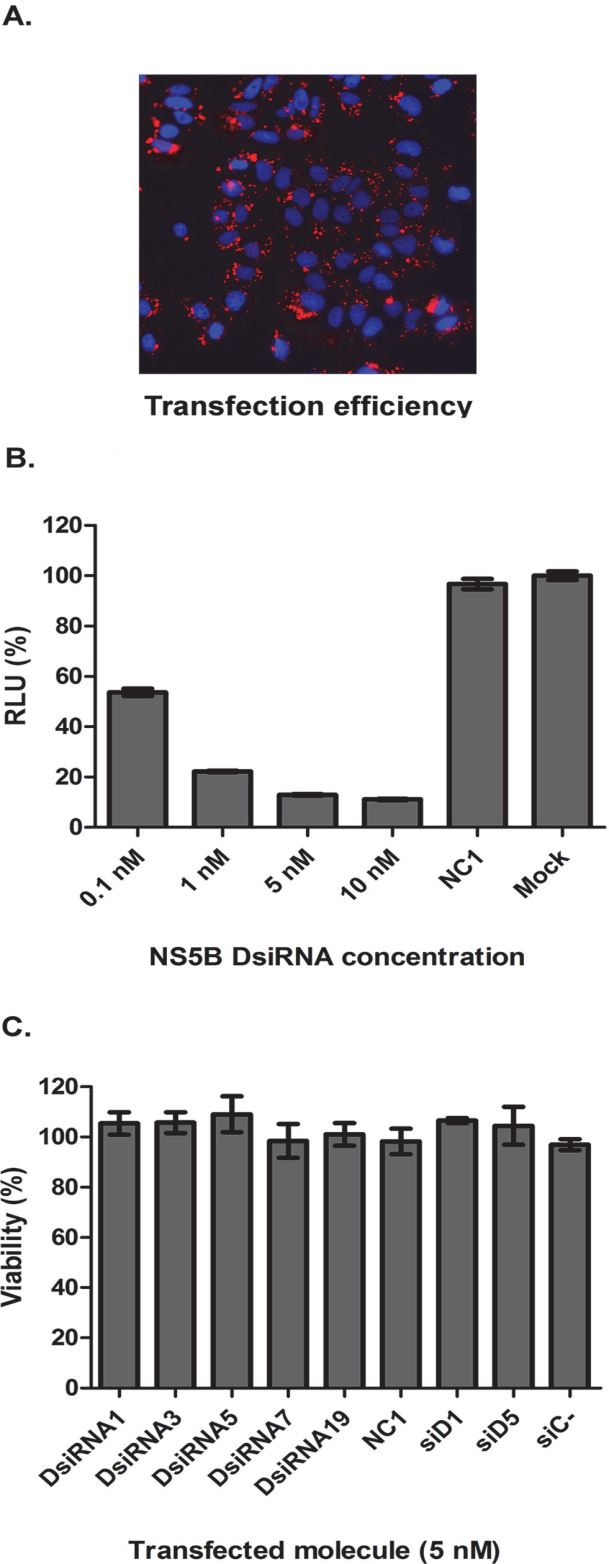
DsiRNA concentration screening. SGR-Feo-JFH-1 cells were transfected with a fluorescent DsiRNA control (5 nM) (A) or with HCV-specific NS5B DsiRNA and 10 nM of DsiRNA negative control (NC1) (B). RNAi molecules do not induce cytotoxicity in SGR-Feo-JFH-1 cells at 5 nM concentration (C). The results are shown as a percentage of the treated sample compared to the mock control (sample transfected with media only). The data (B and C) are averages of three technical replicates and at least two biological replicates, error bars are SD.

The parameters for the efficient transfection of Huh7.5 cells with DsiRNA were established using a fluorescent DsiRNA—the transfection efficiency routinely ranged from 85 to 90% (Fig. 1A). Using the same transfection protocol with varying concentrations of DsiRNA in cells stably harbouring the SGR-Feo-JFH-1, we observed that the DsiRNA directed against the NS5B coding region could reduce the virus replication, as assessed by a luciferase assay (Fig. 1B). As expected, the decrease in luciferase depended on the concentration of DsiRNA used in the transfection. Concentrations as low as 0.1 nM could reduce the levels of luciferase by more than 40% compared to the negative control. The most potent inhibition was observed using 10 nM of DsiRNA; however, the difference between this value and the value obtained at 5 nM was small (<5%). Therefore, the latter concentration was used in all subsequent experiments. To confirm the specificity of the inhibition, the cells were transfected with a negative control DsiRNA (IDT). Similar luciferase levels were observed between cells treated with negative control DsiRNA or mock transfected cells (transfection reagents only), which confirmed that the reduction of luciferase levels using virus-directed DsiRNAs was specific and not an off-target effect or a cell response to transfection (Fig. 1B). To check the cytotoxicity of the RNAi molecules, the cells were transfected with 5 nM of the five DsiRNAs, the two canonical siRNA and their negative controls. After 48 hours, cellular cytotoxicity was not observed for any of the analysed molecules (Fig. 1C).

**Fig 2:**
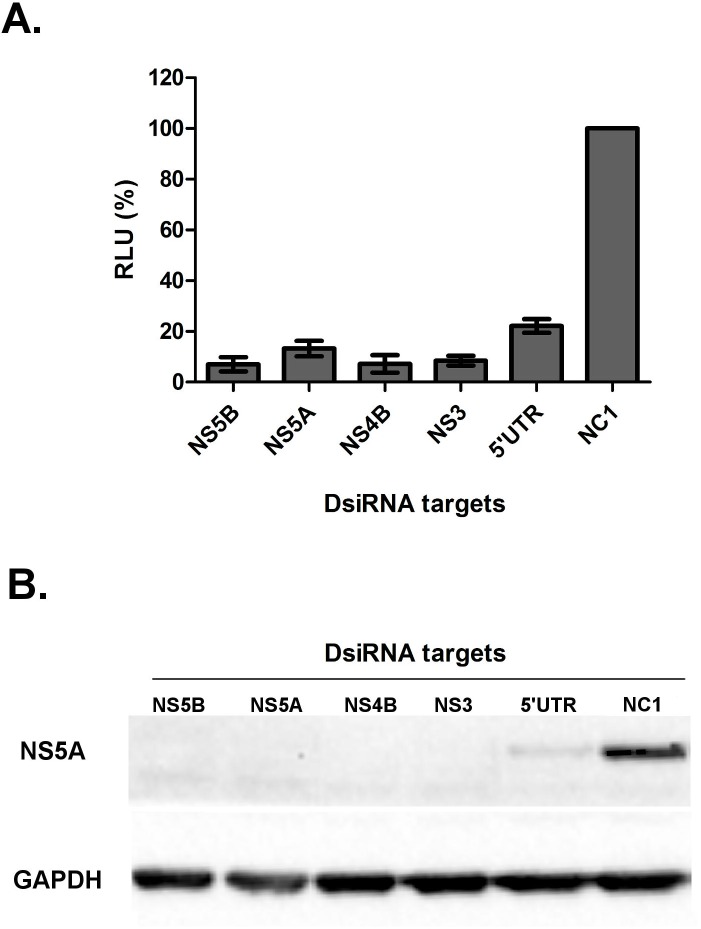
HCV knockdown by different DsiRNAs. SGR-Feo-JFH-1 cells were transfected with 5 nM aliquots of each DsiRNA and analysed 48 h post-transfection by measuring the luciferase levels (A) lysates and protein expression levels (B) in cell lysates. The values are a percentage of the luciferase activity relative to the negative control (NC1) DsiRNA-transfected cells. The labels on the X-axis indicate the region to which DsiRNAs were directed. The data are averages of three technical replicates and at least two biological replicates, error bars represents SD.

We then sought to determine whether different DsiRNAs inhibited HCV replication with similar efficiency. As shown in Fig. 2A, DsiRNAs that targeted different regions of the viral genome inhibited HCV replication to differing extents. Forty-eight hours after transfection, all of the DsiRNAs tested reduced HCV replication (Fig. 2) by at least 77%, with the most efficient DsiRNA targeting the NS5B coding sequence (93% ± 2.8 reduction compared to negative control), followed by NS4B (92.8% ± 3.4), NS3 (91.6% ± 1.9), NS5A (86.8% ± 3.1) and 5’ UTR (77.9% ± 2.7). To confirm this result, the expression of the NS5A protein was evaluated with a western blot assay using a polyclonal NS5A antiserum. Four of the five DsiRNAs reduced the expression of NS5A to undetectable levels, and NS5A was only detected at a low level in cells transfected with the DsiRNA targeting the 5’UTR (Fig. 2B).

**Fig 3:**
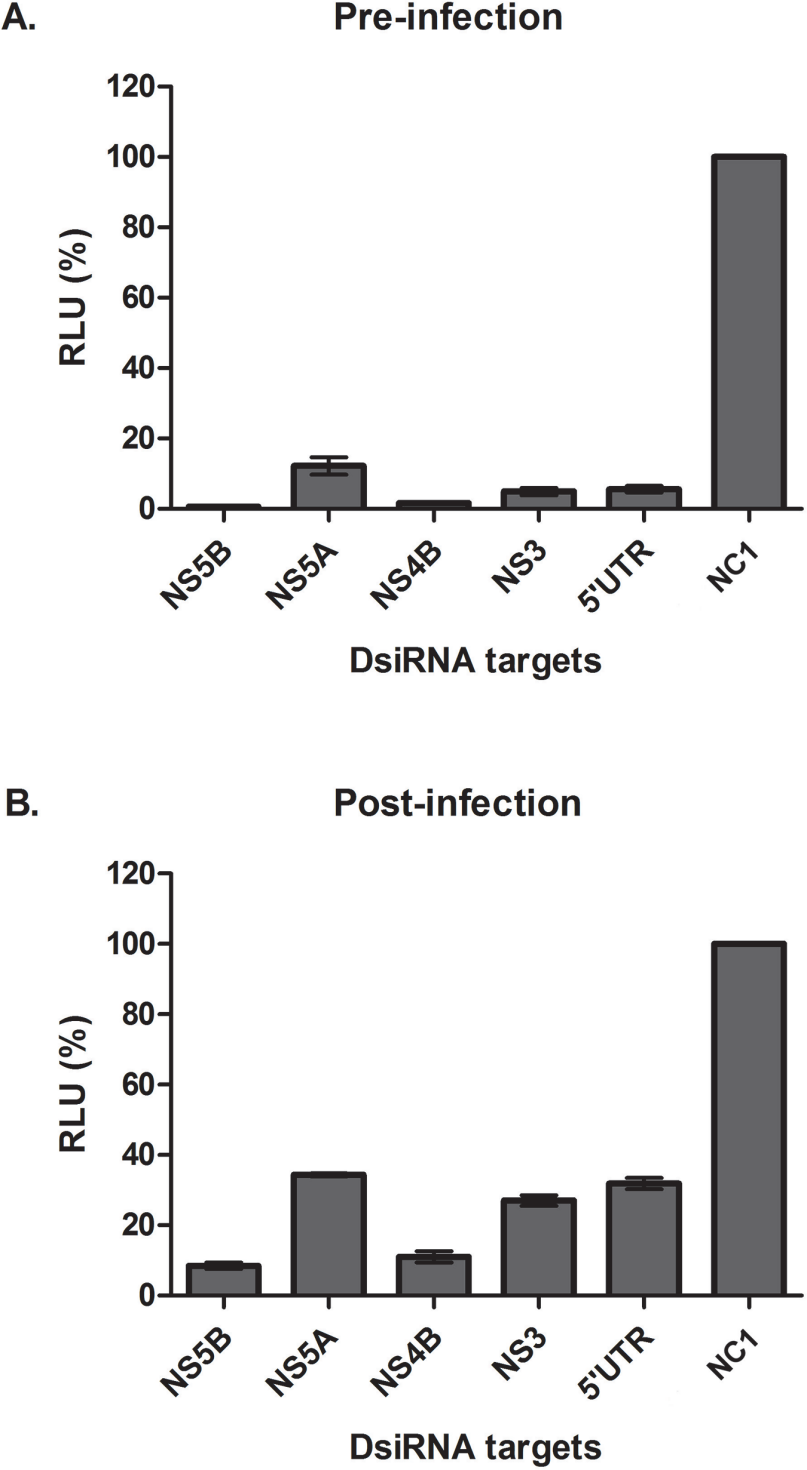
Inhibition of HCVcc by DsiRNAs. Huh7.5 cells were transfected with 5 nM of each DsiRNA before (A) or after (B) infection with HCVcc (MOI of 0.1). At 48 h post-infection, the cells were lysed in PLB and the luminescence levels were read on a BMG plate reader. The values are a percentage of the luminescence of treated sample compared to the negative control (NC1). The data (A and B) are averages of three technical replicates and at least three biological replicates, error bars represents SD.

To assess whether the DsiRNAs molecules were also effective in the context of virus infection, the cells were transfected with each of the five DsiRNAs 24 h prior to infection with FL-J6/JFH-5′C19Rluc2AUbi (HCVcc). At 48 h after infection, renilla luciferase activity was reduced by more than 90% for all DsiRNAs, with NS4B- and NS5B-targeting DsiRNAs almost completely abrogating HCV RNA replication (Fig. 3A). Depending on the DsiRNA tested, the virus replication measured based on the intracellular Renilla luciferase levels was reduced by more than 99.5% (NS5B) and 98.4% (NS4B). In comparison, when cells were infected prior to transfection, the inhibition levels were similar to those observed in the sub-genomic replicon system (Fig. 3B). The two most efficient molecules were those that targeted the coding sequence of NS5B (92.5%) and NS4B (90.6%).

**Fig 4:**
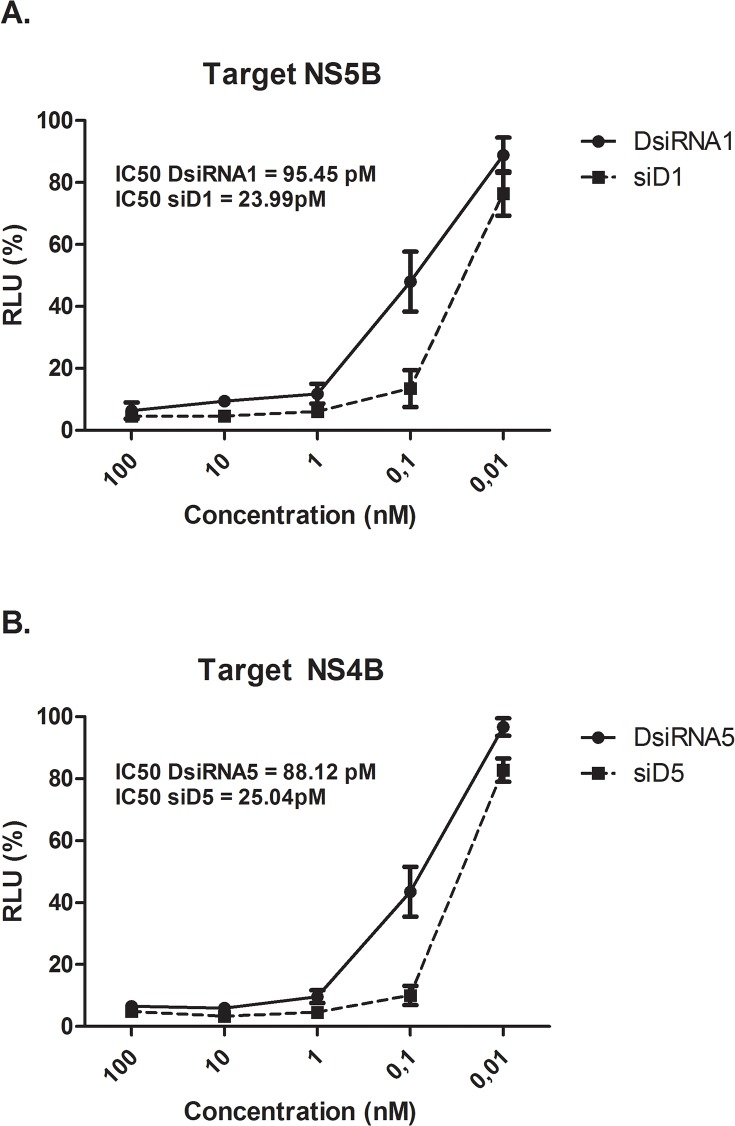
Potency comparison between DsiRNAs and 21 nt siRNAs. SGR-Feo-JFH-1 cells were transfected with the indicated concentrations of DsiRNA or canonical siRNA for two distinct targets. At 48 h post-infection, the cells were lysed in PLB and the luminescence levels were read on a BMG plate reader. The values are a percentage of the luminescence of the treated sample compared to the negative control (NC1). The data (A and B) are averages of three technical replicates and at least three biological replicates, error bars represents SD.

Some studies have reported that DsiRNA molecules are more potent than the corresponding 21-nt siRNA [18,20]. To test this hypothesis, we selected the two most potent DsiRNA molecules (NS5B and NS4B targeted, DsiRNA1 and DsiRNA5, respectively) in our study and synthesised conventional siRNAs (SiD1 and siD5) to the same HCV genome localisation (Table 1). The two types of RNAi molecules were tested in parallel by transfecting SGR-Feo-JFH-1-harbouring cells with concentrations ranging from 0.01 nM to 100 nM. The highest concentrations (> 1 nM) of both molecules (directed to the NS5B region (Fig. 4A) or NS4B region (Fig. 4B)) did not result in differences in inhibition (p>0.05) after incubation for 48 hours. However, at higher dilutions (0.01 nM and 0.1 nM), the canonical siRNAs were more efficient (P <0.05) in reducing HCV replication. To elucidate the difference in the potency of these molecules, we calculated their IC_50_ values. The IC_50_ values calculated for DsiRNA1 and siD1 were 95.45 pM and 23.99 pM (p < 0.001), respectively. For molecules targeting the NS4B region, the IC_50_ values were 88.12 pM for DsiRNA 5 and 25.04 pM for siD5 (p < 0.001).

**Fig 5:**
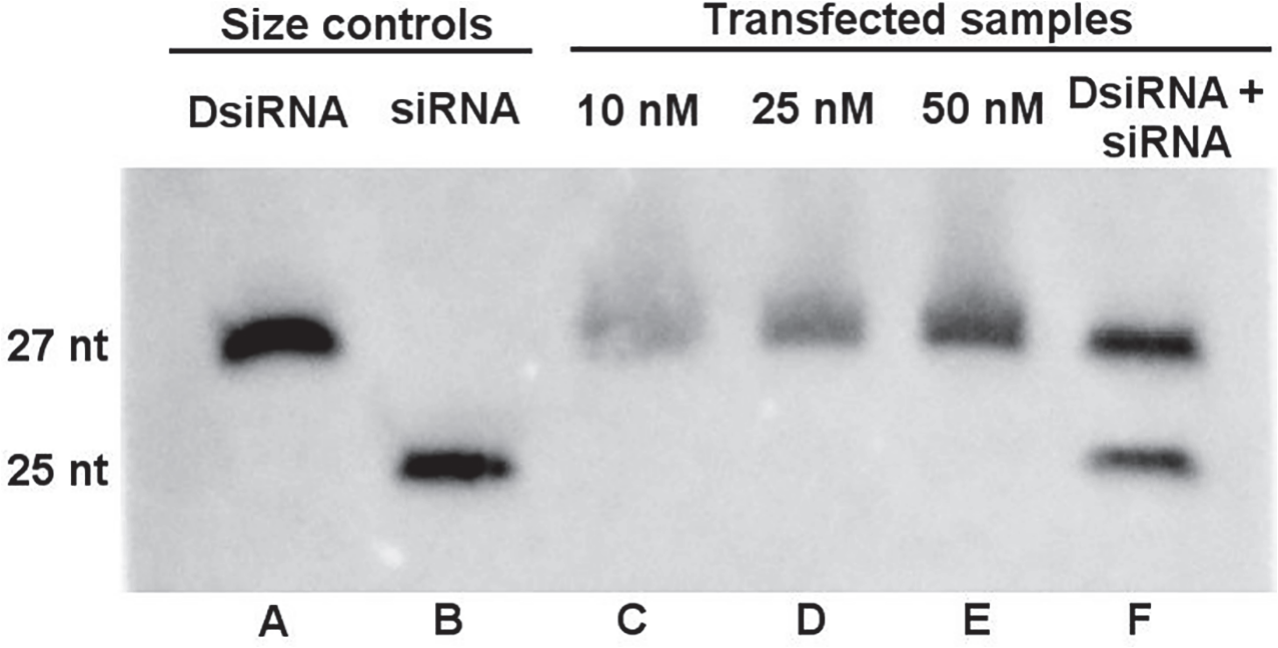
Detection of transfected RNAi by northern blot. Huh7.5 cells were transfected with different concentrations of DsiRNA 1 after 16 hours cells were harvested and RNA extracted and tested by northern blot for the presence of DsiRNA1 and its 21 nt DICER processed form. Untransfected DsiRNA 1 (27 nt) (A) and siD1 (21 nt) (B) were utilized as size controls. Lanes C, D and E Huh7.5 cells transfected with 10 nM, 25 nM and 50 nM respectively. Lane F Huh7.5 co-transfected with DsiRNA1 and siD1 at 25 nM.

DsiRNAs molecules are predicted to be processed by DICER endonuclease into smaller fragments that are then incorporated by RISC complex and used as template for specific destruction of complementary targets. To verify that molecules were being processed by DICER, DsiRNA 1 was transfected at different concentrations into Huh7.5 cells and total RNA was extracted from these cells 16 hours post-transfection. The presence of the 27 nt and the predicted 21 nt processed form was analyzed by northern blot using an specific biotinylated probe (Table 1). The sensitivity of this assay was estimated at 80 fmol using synthetic 27 nt and 21 nt molecules (data not shown). We were able to detect the 27 nt form of the DsiRNA molecule on all transfected concentrations (10 nM, 25 nM and 50 nM) however we did not detect the 21 nt DICER processed form at any of the tested concentrations (Fig. 5), suggesting that DICER processing of DsiRNA was not occurring. To confirm that the probe was capable of detecting the 21 nt processed form, Huh7.5 cells were also co-transfected with the 21 nt molecule, as shown at Fig. 5, both molecules were detected by the Northern blot assay.

**Fig 6:**
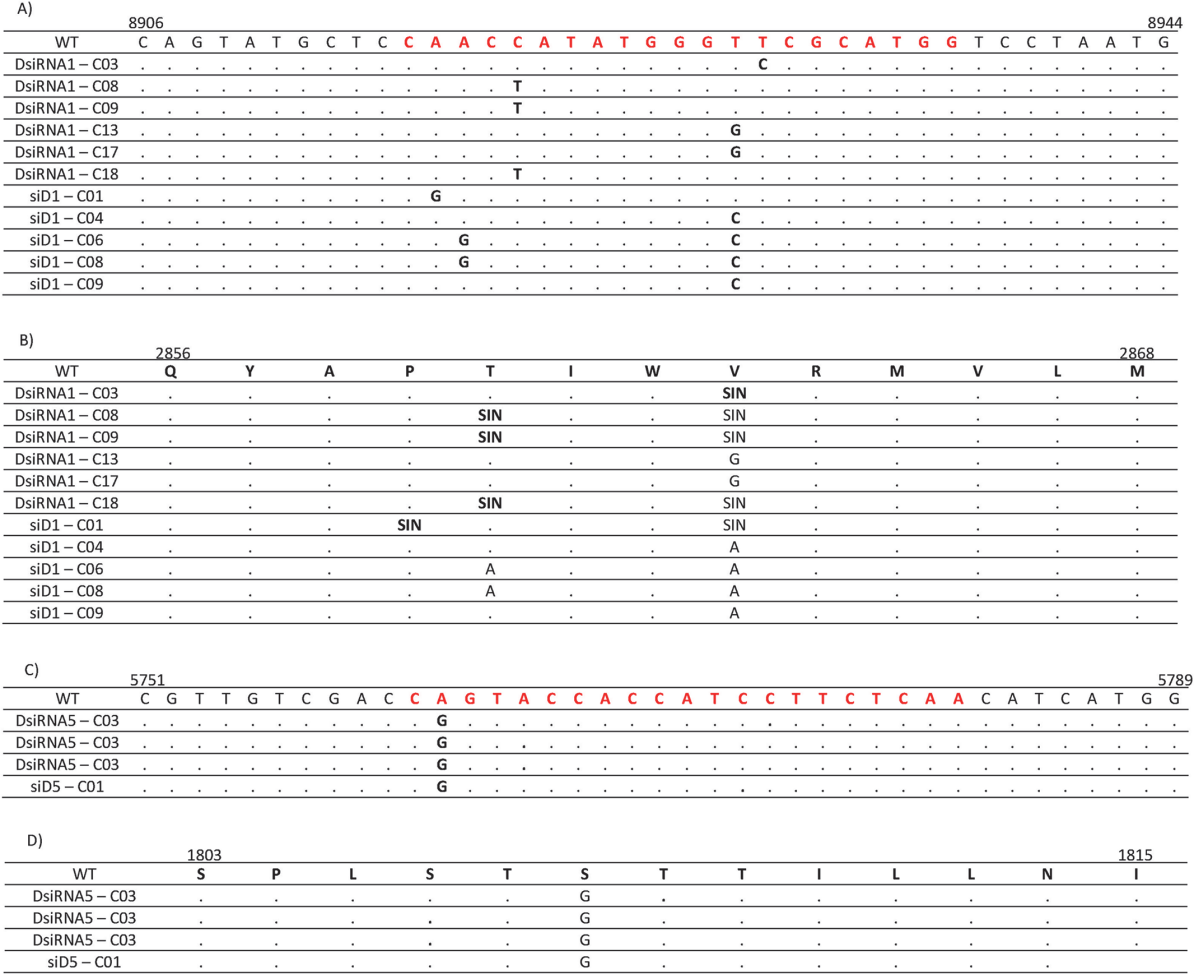
Nucleotide and amino acid sequences of RNAi-resistant cells. The localisation of each mutation observed in siRNA/DsiRNA resistant viral clones. SGR-Feo-JFH-1 cells were repeatedly transfected (5-day intervals) with 5 nM of siD1/siD5 or DsiRNA1/DsiRNA5 and treated with 1 mg/mL of G418. Twenty-one days after the first transfection, the total RNA of surviving colonies was extracted, amplified by RT-PCR and submitted for sequencing. A) Nucleotide substitutions for NS5B target site. B) Amino acid substitutions for NS5B target site. C) Nucleotide substitutions for NS4B target site. B) Amino acid substitutions for NS4B target site. SIN—Synonymous mutations; WT—JFH-1 wild type; Numbers after the RNAi molecule name on first column represents the clone in which sequences were obtained.

The selection of mutants resistant to treatment with RNAi molecules is a common occurrence for RNA viruses. We tested whether the frequency of this phenomenon is similar for DsiRNAs and canonical siRNAs. To this end, SGR-Feo-JFH-1 cells were transfected with 5 nM of either DsiRNA1 or DsiRNA5 (NS5B or NS4B) and their 21 nt corresponding (siD1 or siD5) and treated with G418 for up to 21 days. RNA was extracted from surviving colonies, reverse transcribed, amplified by PCR, cloned and sequenced. When the cells were treated with DsiRNA targeting the NS5B region, 6 of 19 clones containing mutations within the target site were identified. When cells were treated with the corresponding siRNA, four of the 19 clones had mutations in the target region for this molecule. A considerably smaller number of resistant clones was observed for molecules directed to the NS4B region. Of the 15 clones obtained from cells treated with DsiRNA5, 3 (3/15) had mutation at this target site. However, when cells were transfected with siD5, only one (1/15) mutated clone was observed. Finally when cells were treated with the negative controls, none of the 15 clones showed any change in the target site. The nucleotides/amino acid changes, as well as the exact position of these changes, are summarised in Fig. 6.

## Discussion

In this report, we show the ability of Dicer substrate siRNAs (DsiRNAs) to inhibit HCV genome replication *in vitro* using both a sub-genomic and a full-length replication system. In both models, viral replication was reduced by at least 90% using 5 nM of DsiRNAs. To our knowledge, this report is the first to demonstrate the inhibition of HCV by these RNAi molecules. We also demonstrated that unlike other studies [18,20], DsiRNA has similar or even lower inhibition potency as 21 nt siRNAs in the context of HCV.

Off-target effects are a major concern in RNAi therapy [31]. These effects occur when a siRNA is processed by the RNA-induced silencing complex (RISC) and alters the expression of undesirable genes [32]. One of the main processes leading to off-target effects is the siRNA concentration, which is directly proportional to the intensity of undesired effects [31,33]. In general, concentrations near 100 nM are reportedly capable of eliciting non-specific effects [32]. Because DsiRNA is described as a more potent molecule compared to other RNAi molecules [18], a lesser amount of RNA may be used to achieve the desired levels of inhibition, which reduces the likelihood of off-target effects. For this reason, we decided to test these molecules for their ability to inhibit HCV.

We designed five DsiRNA molecules for different regions of the HCV genome. Three molecules that targeted the NS5B, NS4B and NS3 coding regions could reduce viral replication by more than 80%. The other two (5’ UTR and NS5A) molecules only moderately knocked down the virus (< 70%). Although some studies have found that virus replication may be reduced using siRNAs directed to the 5’UTR [34,35] or NS5A [16,36], our DsiRNA molecules targeting those regions were not very efficient. Specifically, the low efficiency of the 5’UTR-targeted sequence is likely due to the highly structured nature of this site [37], which may hinder the binding of the DsiRNA molecule to its target sequence.

After the success of experiments with the sub-genomic system, we tested the efficacy of the DsiRNA molecules in the context of virus infection. In this assay, two different approaches were tested. First, Huh7.5 cells were transfected with DsiRNAs prior to infection with HCVcc, as expected, the levels of inhibition using this approach were much better than those observed when cells were with HCVcc 24 h prior to transfection with DsiRNAs. Overall, the levels of inhibition when cells were infected prior to transfection were similar to those observed in the assays with the sub-genomic system and described by other authors [35,38]. When directly comparing the efficiency of the DsiRNAs against sub-genomic replicons or virus infection, our data suggest that DsiRNAs are less effective once virus infection has been established than in cells harbouring pre-existing subgenomic replicons. The presence of the core protein may protect the nascent virus RNA from DsiRNA-mediated cleavage as a result of associating with the virus RNA during the process of virion assembly.

Some studies showed that DsiRNA molecules were more potent in inhibiting mRNA or viral RNA compared to canonical 21 nt siRNA [18,20]. To test this hypothesis, we synthesised two siRNAs (21 nt) that utilised the same target used by DsiRNA1 or DsiRNA5 (termed siD1 or siD5, respectively). We did not observe any difference in the inhibition potency between siD1 and DsiRNA1 or between siD5 and DsiRNA5 at higher concentrations (1–100nM). Moreover, 21 nt siRNAs more efficiently inhibited virus replication at lower concentrations (0.01–0.1 nM). In a similar study, Foster et al. [39] compared the efficiency of several siRNA and DsiRNA for two human mRNA and also did not observe any difference between these two RNAi molecules. This contradictory information suggests that the increased potency of DsiRNAs may be due to differences in the methodology and experimental models used by the other authors [18,20]. Therefore, we can only affirm that siRNA and DsiRNA have similar potency for HCV inhibition.

The two critical players in the RNAi pathway are DICER, an endoribonuclease responsible for cleaving long (> 21 nt) double-stranded RNAs into smaller pieces with 21 nt of extension, and the RISC complex, which is responsible for unwinding one dsRNA strand and using it as a template to identify homologous mRNAs in order to cleave them [14]. The higher potency of DsiRNA molecules may be the result of a previously identified link between DICER and the RISC complex [40,41]. Previous studies [28] have demonstrated that physical parameters of the DsiRNA molecules (e.g., asymmetry of the molecule and the addition of DNA bases in sense strand) induce DICER to cleavage larger RNA molecules at an exact point, resulting in only a single shorter 21 nt variant. Because we did not observe differences in the inhibition efficiency between the 21 nt and 27 nt molecules, we decided to test if the DsiRNA 1 molecule was being authentically processed by DICER. Although this molecule was designed using a software using the algorithm described by Kim et al. (2005), Northern blot analysis demonstrated that this molecule was not being processed in the predicted manner.

However, DsiRNA1 was still able to inhibit HCV replication efficiently, reaching levels similar to a conventional siRNA molecule. Previous studies have shown that the RISC complex is able to incorporate larger ssRNA molecules (> 21 nt) with no change in target inhibition efficiency [42]. There are extreme cases in which molecules up to 63 bp were incorporated in RISC complex independently of processing by DICER [43] and still effectively inhibited the target RNA. It seems likely therefore that in some cases, DsiRNAs can be directly incorporated into the RISC complex regardless of their processing by DICER and can then act as the guide strand to target viral RNA.

Importantly, we obtained a similar level of inhibition using a 20-fold lower concentration of siRNA when comparing the inhibition efficiency of the siD1 molecule with other studies that used the NS5B HCV coding sequence as a target [44–47]. Similar results were observed for siD5, which was 5 times more potent than the siRNA that targeted the NS4B coding region used in other studies [48]. The algorithm used to predict DsiRNA molecules was first described by Kim et al. [18] and differs at some points from those used to predict siRNA molecules [49–51]. In both cases, the algorithm considers several factors, including the incorporation of transfected dsRNA into the RISC complex and the characteristics of the target region. Both siD1 and siD5 were designed from the corresponding DsiRNA sequences and therefore followed the published protocol for DsiRNAs [18]. This algorithm may more efficiently predict factors that are not taken in account by other prediction software, which allows it to suggest more potent molecules.

A well-known characteristic of HCV replication is the rapid generation of virus variants. A single patient contains many variants of the initial infectious sequence, which are referred to as a quasi-species [52]. These variants are the result of the high error rate of the RNA-dependent RNA polymerase [21]. To be effective, an RNAi molecule needs to be 100% homologous to its target sequence. Consequently, a single base change to the target sequence of a siRNA or DsiRNA could greatly reduce the efficiency of these molecules [53]. Thus, the elevated error rate of HCV replication may be an issue during treatments with RNAi molecules, as reported by Konishi et al. [22].

As demonstrated by Northern blot, DsiRNA1 was not processed to the predicted 21 nt product and as a result a single 27 nt molecule might have been incorporated into the RISC complex [42]. Therefore the viral RNA target site for both molecules (DsiRNA 1 and siD1) were almost identical, except for the extra 6 nt for the larger molecule and this fact may justify the similarity of the number of resistant colonies obtained, when these molecules were compared. Also the number of resistant colonies without on-site mutations after the treatment was relatively high and this may be explained by the transfection efficiency. Although our efficiency was higher than 90%, even after 3 transfections a number of cells might remain untransfected. Consequently, those untransfected/poorly transfected cells could survive the G418 treatment, to develop colonies that were later collected and sequenced.

Moreover, the presence of mutations outside the target of RNAi molecules caught our attention. In addition to the clones identified with mutations in the NS5B or NS4B targets, we observed few clones with point mutations within the limit of 100 nt 5' or 3' to the original target site. This result was observed for cells treated with all RNAi molecules tested in this study. However, the mutations in each clone were in different positions. These mutations outside the target have been observed in the context of other viruses [54], and these nucleotide changes were suggested to play a role in the emergence of resistant mutants to treatment with RNAi by a rearrangement in the local structure of RNA, making the target region inaccessible to siRNA/DsiRNA molecules [55]. However, mutations outside the target were also observed in cells treated with the negative control, though these nucleotide changes were different from those observed in cells treated with siRNA/DsiRNA. Therefore, whether these mutations had a role in the RNAi resistance process in our study is unclear.

In this study, we reduced replication of an HCV sub-genomic replicon by more than 90% and the replication of HCVcc virus by approximately 99% using Dicer substrate siRNAs. Contrary to other experimental models, we also showed that DsiRNA have similar potency as a conventional 21-nt siRNA for the inhibition of HCV. Finally, we demonstrated that treating the SGR-Feo JFH-1 replicon for 21 days with any of our RNAi molecules (siRNA or DsiRNA) resulted in similar frequencies of treatment-resistant mutants. Therefore, we conclude that DsiRNA molecules have similar potency as 21 nt siRNAs for the inhibition of HCV genome replication and should be considered as part of future HCV therapy.
